# Cardiovascular screening prior to stem cell transplantation in the United Kingdom

**DOI:** 10.1002/jha2.599

**Published:** 2022-10-18

**Authors:** David G. Gent, Muhammad Saif, Julia Lee, Arpad G. Tóth, Eleni Tholouli, Rebecca Dobson, David J. Wright

**Affiliations:** ^1^ Liverpool Centre for Cardiovascular Science University of Liverpool and Liverpool Heart and Chest Hospital Liverpool UK; ^2^ The Clatterbridge Cancer Centre Liverpool UK; ^3^ British Society of Blood and Marrow Transplantation and Cellular Therapy Data Registry London UK; ^4^ Department of Haematology Manchester University NHS Foundation Trust Manchester UK

**Keywords:** cardiovascular screening, hematopoietic cell transplantation, stem cell transplantation

1

Haematopoietic cell transplantation (HCT) recipients experience an increased risk of cardiovascular disease compared to the general population[1, 2]. Although transplant‐related mortality has decreased over the last decades, this has not been through a reduction in death due to cardiovascular disease [3, 4]. Improving cardiovascular outcomes post‐transplant is a focus of the International Cardio‐Oncology Society [5], and the European Society of Cardiology has recently produced guidelines for risk assessment and surveillance [6].

Given this background, we aimed to investigate the current United Kingdom (UK) and Republic of Ireland (ROI) pre‐transplant cardiovascular screening practice. We wanted to understand if screening was performed, which investigations were used and if a left ventricular ejection fraction (LVEF) cut‐off was an exclusion criterion for HCT. We also wanted to investigate what cardiology support was available to transplant centres, and if there were dedicated cardio‐oncology services. This study surveyed adult and paediatric units.

A 23‐item survey was created with three introductory questions and 13 adult and seven paediatric‐specific questions (Supplementary Material). Centres could answer both sections, and they had the option to skip questions. The survey was distributed as an internet‐based questionnaire (surveymonkey.com) through the British Society of Blood and Marrow Transplantation and Cellular Therapy to transplant directors at the 52 UK and ROI transplant centres. The study was open from March 2022 to June 2022, and three reminders were sent out to non‐respondents before the survey closed.

A total of 26 centres responded (50% response rate) without duplicates. One adult centre erroneously answered the paediatric section giving 25 complete responses. Response rates were equal across England, Wales and Scotland; however there were no responses from either Northern Ireland or the ROI. Fifty‐five percent (18/33) of the centres that perform both autologous and allogeneic transplants and 42% (8/19) of the centres that perform autologous only transplants responded. From the responding centres, 21 only performed adult transplants, three exclusively performed paediatric transplants, and two performed combined adult and paediatric transplants.

Over three quarters of the adult centres (76%) recorded a co‐morbidity score prior to transplant; the majority used the hematopoietic cell transplantation co‐morbidity index [7]. This score has 17 sections, with three corresponding to cardiac disease. Despite not all centres using a co‐morbidity score, they all performed some form of cardiovascular screening. One centre only preformed routine screening for patients prior to allogeneic transplants, not autografts (Table 1a).

**TABLE 1:** Cardiovascular screening practice in UK transplant centres

**a. Adult responses**	
***Do you record a co‐morbidity score? (n = 21)***	% (*n*)
Yes	76% (16)
No	24% (5)
Which score? (n = 16)*	% (*n*)
Haematopoietic cell transplantation co‐morbidity index (HCT‐CI)	94% (15)
Age adjusted HCT‐CI	6% (2)
Charlson co‐morbidity index	0% (0)
Pre‐transplant assessment of mortality (PAM) score	0% (0)
Other	0% (0)
*One centre selected two scores	
***Do you perform cardiovascular screening on all patients?***	
Combined allogeneic and autologous centres (*n* = 14)	% (*n*)
Yes	93% (13)
No	7% (1)*
*This centre did, however, do cardiovascular screening on all patient having an allogeneic transplant	
Autologous only transplant centres (*n* = 7)	% (*n*)
Yes	100% (7)
No	0% (0)
***Which investigations do you use? (n = 21)***	% (*n*)
Trans‐thoracic echocardiography	90% (19)
12‐lead ECG	86% (18)
Cardiac CT, MRI or nuclear medicine scanning	33% (7)
Serum biomarkers (BNP and/or troponin)	19% (4)
Cardio‐pulmonary exercise testing	19% (4)
Other	0% (0)
***Do you have an LVEF cut‐off value for transplant candidate selection? (n = 19; two adult centres did not answer)***	% (*n*)
Yes	81% (13)
No	19% (6)
***Do you refer select patients to cardio‐oncology services after stem cell transplant? (n = 19)***	% (*n*)
Yes ‐ all patients	0% (0)
Yes ‐ select patients	32% (6)
No	68% (13)
***Do you have criteria for cardio‐oncology referral? (n = 19)***	% (*n*)
Yes	26% (5)
No	74% (14)
**b. Paediatric responses**	
***Do you perform cardiovascular screening on all patients? (n = 5)***	% (*n*)
Yes	100% (5)
No	0% (0)
***What does your cardiovascular screening involve? (n = 5)***	% (*n*)
Co‐morbidity score	20% (1)
12 lead ECG	20% (1)
Trans‐thoracic echocardiography	80% (4)
Other	0% (0)
***Do you have criteria for cardio‐oncology referral? (n = 5)***	% (*n*)
Yes	60% (3)
No	40% (2)
***Are all patients referred for cardio‐oncology follow up? (n = 5)***	% (*n*)
Yes	0% (0)
No	100% (5)
***Are patients referred for late‐effects clinic follow up? (n = 5)***	% (*n*)
Yes	100% (5)
No	0% (0)

Abbreviations: ECG, electrocardiogram; HCT, haematopoietic cell transplantation.

A variety of investigations were used for adults with most centres using trans‐thoracic echocardiography (TTE; 90%) and a 12‐lead electrocardiogram (ECG; 86%). There was infrequent use of serum biomarkers (19%), more advanced cardiac imaging techniques (33%), and cardio‐pulmonary exercise testing (CPEX; 19%). One centre that had used CPEX stopped at the start of the COVID‐19 pandemic.

Most adult centres (81%) used an LVEF measurement to exclude patients from transplantation. There was heterogeneity in the value used for exclusion, ranging from ≤50% to ≤30% with no justification for the difference. Only one centre that used ≤40% as an exclusion criterion specified that they use reduced intensity conditioning (RIC) if the LVEF is ≤50% (Figure 1).

**FIGURE 1:**
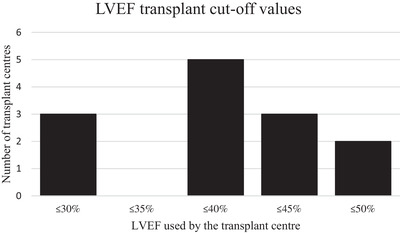
**The spread of LVEF values used as a boundary for transplant exclusion**. The figure shows the different LVEF values used as a cut‐off and the number of centres using each value. Only one centre specified that, although they used ≤40% as a boundary, if the LVEF was ≤50% a reduced intensity conditioning regimen was used

Post‐transplantation, 32% of adult centres referred select patients for cardio‐oncology review, and a quarter of centres had specific referral criteria. In general, patients were referred on a case‐by‐case basis if there were clinical concerns or a low LVEF. There was substantial variation in cardiology support; some centres found it difficult to get cardiologists interested, whereas other units had dedicated cardio‐oncology clinics. In the most well‐defined service, all patients with a history of ischaemic heart disease, heart failure or anthracycline‐related cardiomyopathy were referred for cardiology‐oncology consultation with a stress echocardiogram or cardiac MRI, and where possible all patients over 60 years were referred for clinical review. If patients had an impaired LVEF the most common pre‐transplant pharmacotherapy used was a beta blocker and angiotensin converting enzyme inhibitor combination.

For paediatric centres (Table 1b), all children who had HCT received some form of cardiovascular screening. The majority (80%) had LVEF assessed by TTE, and a small proportion had a 12‐lead ECG or co‐morbidity score. Sixty percent of the paediatric centres had criteria for cardio‐oncology referral. Although children were not routinely referred for cardio‐oncology follow up, all were referred to the late effects clinic.

The heterogeneity in the LVEF boundary for adults was surprising (Figure 1). The majority of assessment was performed using TTE, which is associated with significant intra‐ and inter‐observer variability [8]. Previous studies do not support a LVEF boundary cut‐off and suggest patients with a moderately impaired LVEF can safely undergo HCT, particularly with reduced toxicity regimens [9, 10]. There is limited data for patients with severely impaired LVEF (≤35%). The number of centres selectively using RIC may be higher than that captured by this survey. Given the effects of HCT on the haematological and pulmonary systems, a more global assessment of cardiovascular fitness pre‐transplant with CPEX may have a greater ability to predict adverse outcomes post‐transplant, although this needs further research [11, 12].

Other significant knowledge gaps include the utility of blood and imaging biomarkers on long‐term risk stratification. Although recommendations on cardiovascular risk assessment and ongoing surveillance exist, the majority are formed from expert consensus [6, 13, 14] . A cardiovascular risk score for the prediction of heart failure and coronary artery disease 10 years post‐transplant has been generated using variables available one year after transplant (history of anthracycline exposure, chest radiation, hypertension, age, diabetes, and smoking)[15]. Despite this, the optimal approach to monitoring and treating patients at different levels of risk is unknown, and there are little data to help us predict short term complications arising 100 days to 6 months after HCT in both adult and paediatric patients.

The results of our analysis provide an overview of pre‐transplant cardiovascular screening practice in the UK, and the response rate provides an acceptable sample of transplant centre activity. The marked inter‐site variability in adult practice reflects the historical absence of guidelines to help centres select patients for pre‐transplant screening; however given the publication of recent recommendations, there may be a more uniform approach in the future.

## AUTHOR CONTRIBUTIONS

David G. Gent wrote the survey, analysed the responses and drafted the manuscript, table and figure. Muhammad Saif, Julia Lee, Arpad G. Toth, Eleni Tolouli, Rebecca Dobson and David J. Wright reviewed and edited the survey and the manuscript. Julia Lee disseminated the survey and prompted to the transplant centres. David G. Gent, Rebecca Dobson and David J. Wright came up with the concept.

## CONFLICT OF INTEREST

Professor Wright has received consultancy and speaker fees from Boston Scientific and Medtronic outside the submitted work. All other authors have no relationships relevant to the content of this paper to disclose.

## Supporting information

Supporting Information

## Data Availability

The dataset gathered in this study is available from the corresponding author on request.
