# Supplementary material for: Cardiovascular screening prior to stem cell transplantation in the United Kingdom
Source: EJHaem. 2022 Oct 18;3(4):1455–8. doi: 10.1002/jha2.599 (PMC9713214; doi:10.1002/jha2.599)
Supplement: Supplementary file 1 — Supporting Information [file JHA2-3-1455-s001.docx]

Dear Colleague

We are surveying UK cardiovascular screening practice prior to stem cell transplantation (for all transplant indications in adults and children).

Please could you take the time to answer the following survey:

1. ***What is your job title?***

|  |
| --- |

1. ***Which institution do you work at?***

|  |
| --- |

1. ***Do you predominantly deal with adult or paediatric patients?***

| **Adult** |  |
| --- | --- |
| **Paediatric** |  |

If **adult** patients please answer part A (age 18 onwards)

If **paediatric** patients please answer part B (age below 18)

Please answer both sections if you deal with both paediatric and adult patients

**Part A – Adults**

1. ***Do you perform cardiovascular screening on all patients prior to stem cell transplantation?***

| Yes |  |
| --- | --- |
| No |  |

If yes, which of the following modalities do you use (please tick all boxes applicable):

| 12-lead ECG |  |
| --- | --- |
| Serum biomarkers (BNP/NT-proBNP and/or Troponin I or Troponin |  |
| Trans-thoracic echocardiography |  |
| Cardiac CT, Cardiac MRI or Nuclear medicine cardiac scanning |  |
| Metabolic Cardio-Pulmonary Exercise Testing |  |
| Other | |

1. ***Do you assess and document comorbidity score for HSCT patients:***

| Yes |  |
| --- | --- |
| No |  |

If yes, which score do you use:

| Haematopoietic cell transplantation comorbidity index HCT-CI |  |
| --- | --- |
| Age adjusted HCT-CI |  |
| Charlson comorbidity index |  |
| PAM score |  |
| Other | |

1. ***When do you use comorbidity score:***

| For allogenic HSCT |  |
| --- | --- |
| For autologous HSCT |  |
| For both autologous and allogenic HSCT |  |

1. ***If you do not routinely screen for cardiovascular disease prior to transplantation, do you perform a more focussed cardiovascular assessment in select cases?***

| Yes |  |
| --- | --- |
| No |  |

If yes, which one of the following factors prompt further cardiovascular assessment in your practice (please tick all boxes applicable):

| Significant cumulative dosage of anthracyclines |  |
| --- | --- |
| Thoracic radiotherapy / TBI |  |
| Use of cyclophosphamide / etoposide |  |
| History of coronary artery disease |  |
| History of cardiac arrhythmia |  |
| Presence of diabetes |  |
| Previous diagnosis of hypertension |  |
| Significant family history of cardiac disease |  |
| Extensive smoking history |  |
| Underlying indication for SCT such as haemoglobinopathy |  |
| Other | |

1. ***If you do not do any routine or selective cardiovascular screening do you think that this has a role in transplant pre-assessment work up?***

| Yes |  |
| --- | --- |
| No |  |

If not, please describe the reasons why you don’t think it is needed

|  |
| --- |

1. ***Do you exclude patients from stem-cell transplantation if they have a left ventricular ejection fraction (LVEF) below a certain value?***

| Yes |  |
| --- | --- |
| No |  |

If yes, what value do you use as a cut-off?

|  |
| --- |

1. ***Do you refer all patients to cardio-oncology services post haematopoietic stem cell transplantation?***

| Yes |  |
| --- | --- |
| No |  |
| Allogenic HSCT only |  |

If no, do you have established criteria for referral to a cardio-oncology service?

| Yes |  |
| --- | --- |
| No |  |

**Part B - Children**

1. ***Do you perform cardiovascular screening on all children prior to stem cell transplantation?***

| Yes |  |
| --- | --- |
| No |  |

If yes, what does your screening involve?

| Age adjusted HCT-CI |  |
| --- | --- |
| Charlson comorbidity index |  |
| PAM score |  |
| Other | |

1. ***If you do not routinely screen for cardiovascular disease prior to transplantation do you perform a more focussed cardiovascular assessment in select cases?***

| Yes |  |
| --- | --- |
| No |  |

If no, what specific factors would prompt further cardiovascular assessment in your practice?

|  |
| --- |

1. ***If you do not do any routine or selective cardiovascular screening do you think that this has a role in transplant pre-assessment in children?***

| Yes |  |
| --- | --- |
| No |  |

If not, please describe the reason why you don’t think it is needed

|  |
| --- |

1. ***Do you refer all patients to cardio-oncology services post allogenic haematopoietic stem cell transplantation?***

| Yes |  |
| --- | --- |
| No |  |

If no, do you have criteria for referral to cardio-oncology service?

| Yes |  |
| --- | --- |
| No |  |

If yes, please describe the referral criteria:

|  |
| --- |

1. ***Do you refer children for ongoing review in a late-effects clinic?***

| Yes |  |
| --- | --- |
| No |  |
